# Biomedical Promise of Aspergillus Flavus-Biosynthesized Selenium Nanoparticles: A Green Synthesis Approach to Antiviral, Anticancer, Anti-Biofilm, and Antibacterial Applications

**DOI:** 10.3390/ph17070915

**Published:** 2024-07-09

**Authors:** Eman Jassim Mohammed, Ahmed E. M. Abdelaziz, Alsayed E. Mekky, Nashaat N. Mahmoud, Mohamed Sharaf, Mahmoud M. Al-Habibi, Nehal M. Khairy, Abdulaziz A. Al-Askar, Fady Sayed Youssef, Mahmoud Ali Gaber, Ebrahim Saied, Gehad AbdElgayed, Shimaa A Metwally, Aly A. Shoun

**Affiliations:** 1 Department of Microbiology, College of Science, Mustansiriyah University, Baghdad 14022, Iraq; emanjassim@uomustansiriyah.edu.iq; 2 Botany and Microbiology Department, Faculty of Science, Port-Said University, 23 December Street, Port-Said 42522, Egypt; ahmed.abdelaziz@sci.psu.edu.eg; 3 Botany and Microbiology Department, Faculty of Science, Al-Azhar University, Nasr City, Cairo 11884, Egypt; nashaat_mahmoud@azhar.edu.eg (N.N.M.); mg3666066@gmail.com (M.A.G.); hema_almassry2000@azhar.edu.eg (E.S.); 4 Biochemistry and Molecular Biology Department, College of Marine Life Sciences, Ocean University of China, Qingdao 266003, China; mohamedkamel@azhar.edu.eg; 5 Department of Biochemistry, Faculty of Agriculture, AL-Azhar University, Nasr City, Cairo 11651, Egypt; 6 Microbiology and Immunology Department, Faculty of Pharmacy (Boys), Al-Azhar University, Nasr City, Cairo 11651, Egypt; mahmoudalhabibi@azhar.edu.eg; 7 Microbiology and Immunology Department, Egypt Drug Authority (EDA), (Formerly NODCAR), Giza 12654, Egypt; nehal.khairy@su.edu.eg; 8 Microbiology and Immunology Department, Faculty of Pharmacy, Sinai University-East Kantara Branch, Ismailia 41636, Egypt; 9 Botany and Microbiology Department, Faculty of Science, King Saud University, P.O. Box 2455, Riyadh 11451, Saudi Arabia; aalaskara@ksu.edu.sa; 10 Department of Pharmacology Faculty of Veterinary Medicine, Cairo University, Giza 12211, Egypt; fadyalsalhany@cu.edu.eg; 11 Integrated Molecular Plant Physiology Research, Department of Biology, University of Antwerp, 2020 Antwerp, Belgium; gehad.hegazygadabdelgayed@uantwerpen.be; 12 Microbiology and Immunology Department, Faculty of Pharmacy for Girls, Al-Azhar University, Cairo 11651, Egypt; dr.shimaaabdelrahman@azhar.edu.eg; 13 Microbiology and Immunology Department, Faculty of Pharmacy, El Salehey El Gadida University, El Saleheya El Gadida 44813, Egypt; aly.shoun@sgu.edu.eg

**Keywords:** anticancer activity, *Aspergillus flavus*, antiviral activity, medicinal applications, biosynthesized, selenium nanoparticles

## Abstract

This study utilized *Aspergillus flavus* to produce selenium nanoparticles (Se-NPs) in an environmentally friendly and ecologically sustainable manner, targeting several medicinal applications. These biosynthesized Se-NPs were meticulously characterized using X-ray diffraction (XRD), Fourier-transform infrared (FT-IR) spectroscopy, transmission electron microscope (TEM), and UV–visible spectroscopy (UV), revealing their spherical shape and size ranging between 28 and 78 nm. We conducted further testing of Se-NPs to evaluate their potential for biological applications, including antiviral, anticancer, antibacterial, antioxidant, and antibiofilm activities. The results indicate that biosynthesized Se-NPs could be effective against various pathogens, including *Salmonella typhimurium* (ATCC 14028), *Bacillus pumilus* (ATCC 14884), *Staphylococcus aureus* (ATCC 6538), *Clostridium sporogenes* (ATCC 19404), *Escherichia coli* (ATCC 8739), and *Bacillus subtilis* (ATCC 6633). Additionally, the biosynthesized Se-NPs exhibited anticancer activity against three cell lines: pancreatic carcinoma (PANC1), cervical cancer (Hela), and colorectal adenocarcinoma (Caco-2), with IC50 values of 177, 208, and 216 μg/mL, respectively. The nanoparticles demonstrated antiviral activity against HSV-1 and HAV, achieving inhibition rates of 66.4% and 15.1%, respectively, at the maximum non-toxic concentration, while also displaying antibiofilm and antioxidant properties. In conclusion, the biosynthesized Se-NPs by *A. flavus* present a promising avenue for various biomedical applications with safe usage.

## 1. Introduction

The substantial infectivity and fatality rates linked to dangerous micro-organisms, such as *Mycobacterium tuberclosis* (TB), *S. aureus*, and *SARS-CoV-2*, highlight why these infectious illnesses continue to be a major public health problem worldwide. Modern chemotherapeutic techniques are essential for controlling infectious diseases, but their broad usage frequently leads to decreased treatment efficacy and the rise of drug-resistant forms [1]. Additionally, biofilms formed by multidrug-resistant bacteria tend to be impervious to antibiotics, underscoring the necessity for more potent treatments [2]. Consequently, improving the efficacy of existing treatments for infectious diseases emerges as a critical and urgent challenge for public health worldwide [3]. It is difficult to create novel substances with a variety of properties, such as antibacterial, antiviral, anticancer, antioxidant, and insecticidal properties. Nevertheless, because of developments in nanotechnology, a wide variety of chemicals based on nanoparticles are now available for a variety of medicinal and biotechnological applications [4]. In the biomedical industry, nanoparticles have a variety of applications and are made from several materials, including silver, gold, copper, magnesium, zinc, cadmium, and gadolinium. Their employment is limited by things like high manufacturing costs (as with silver, gold, copper, and magnesium) or their possible negative consequences (as with titanium, gadolinium, and cadmium) [5,6].

Selenium plays an essential role in human health, contributing to the function of selenoproteins, antioxidant defense mechanisms, cell signaling pathways, immune system regulation, and various metabolic activities [7]. Antioxidants are crucial in combating oxidative stress, which is linked to various diseases, including neurodegenerative disorders and cardiovascular conditions [8]. Se-NPs, benefiting from the antioxidant characteristics of selenium, help in maintaining redox balance and provide cellular protection [9]. The surface of these biosynthesized nanoparticles often bears phenolic and flavonoid compounds, enhancing their antioxidant activity [10]. Additionally, both hydrophilic and lipophilic antioxidants, along with enzymatic and non-enzymatic systems, contribute to neutralizing oxidative agents [11]. Research has shown a strong correlation between selenium deficiency and increased rates of cancer, infectious diseases, and cardiovascular conditions [12,13]. Both organic and inorganic forms of selenium are commonly used as food supplements. Nonetheless, despite its therapeutic benefits, there is a relatively narrow margin of safety for selenium dosage; excessive consumption can lead to adverse toxic effects [13]. Recently, Se-NPs have attracted more attention in the biomedical industry due to their outstanding biocompatibility and low toxicity, suggesting significant potential applications [14]. Adults typically require a daily intake of approximately 40–300 mg of selenium from dietary supplements for optimal health. Moreover, studies have indicated that Se-NPs exhibit antiviral properties against a variety of viruses, including influenza and hepatitis B, by inhibiting their replication and transcription processes [15].

Furthermore, Se-NPs have been shown to impact a broad spectrum of pathogenic micro-organisms, including bacterial strains such as *Salmonella typhi*, *Bacillus cereus*, *Listeria monocytogenes*, *E. coli*, *Burkholderia cenocepacia*, *P. aeruginosa*, *S. maltophilia*, and *S. aureus*, as well as fungi including *Candida albicans*, *C. tropicalis*, *C. glabrata*, *A. flavus*, and *A. fumigatus* [16,17,18]. Additionally, Se-NPs have to be effective against various types of insect vectors [19]. Fascinatingly, Se-NPs have demonstrated the ability to specifically target macrophages and modulate macrophage polarization, thereby kick-starting innate immunity for antimicrobial action through the regulation of cytokine production [20]. Likewise, Se-NPs function as immunomodulatory agents, enhancing T cell activation and tumor-associated macrophage modulation to enhance antitumor immune responses and impede tumor development [21]. The immunological characteristics of Se-NPs emphasize their potential as immunomodulatory instruments in pathogen defense and their role in the immunotherapy of infectious diseases [22].

Several methods are used to create Se-NPs, such as chemical, physical, and biological processes. The use of chemical and physical methods is frequently discouraged because of their high costs, possible biosafety risks, the production of hazardous byproducts, and strict synthesis requirements [23]. Consequently, biological synthesis emerges as a preferred alternative, addressing these concerns. This environmentally benign method uses natural compounds generated by a range of biological sources, including plants, bacteria, fungus, yeast, and actinomycetes for the reduction process and then coats the surface of the nanomaterials [24,25]. This process not only enhances the stability of the nanoparticles but also mitigates their tendency to aggregate or form clumps over time. This provides a summary of the synthesis methods and biological activities of Se-NPs, along with recent advances in their application for anti-infection treatments, aiming to support future research in anti-infection therapy.

In this study, Se-NPs were synthesized using an aqueous extract from *A. flavus*. The biosynthesized Se-NPs were characterized through various methods such as UV–visible spectroscopy, XRD, FT-IR, TEM, and dynamic light scattering (DLS). The antimicrobial effectiveness of these nanoparticles was evaluated against a range of pathogenic organisms, encompassing both Gram-positive and Gram-negative bacteria. Additionally, the investigation extended to examining their antibiofilm, antiviral, antitumor, antioxidant, and anti-inflammatory capabilities.

## 2. Results

### 2.1. The Fungal Isolate Identification

**Figure 1:**
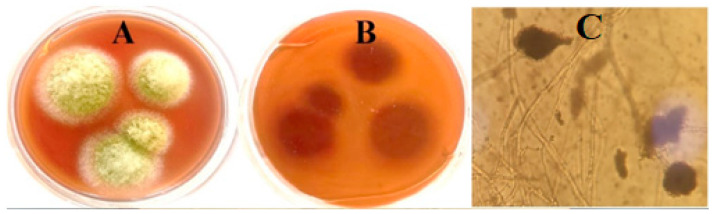
Cultural and microscopic examinaion, of fungal isolate D11. (**A**) Colony of fungal isolate D11 on a potato dextrose agar medium, (**B**) reverse colony of isolate D11 on a potato dextrose agar medium, (**C**) bright-field microscopic examination (X = 800).

According to morphological identification, *A. flavus* colonies usually have a powdery texture with a reddish-gold hue on the bottom and a yellowish-green spore coloring on the top surface. These fungi frequently induce localized discoloration when they infect grains and legumes, giving the affected areas a noticeable dull appearance. These colonies are powdery in substance and develop quickly. The hyphae of the fungal growth are septate and have a transparent (hyaline) appearance. The fungal growth has thread-like branching that creates mycelium (Figure 1).

**Figure 2:**
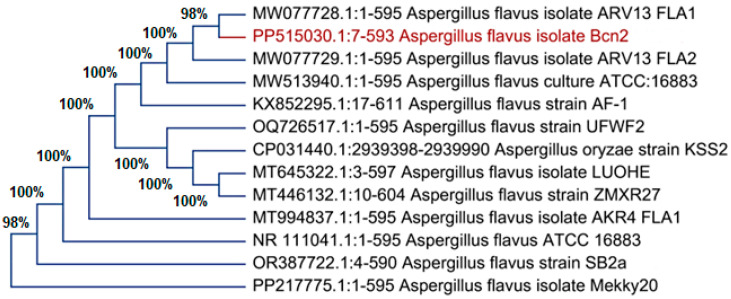
Phylogenetic tree of isolate D11 with the sequences from NCBI. The red text express the name of own isolate *Aspergillus flavus*.

Molecular techniques confirmed the identity of the isolated fungus. Gene sequencing analysis showed that the isolate shares 98% similarity with *A. flavus*, according to its accession number PP217775. This confirmed *A. flavus* as the specific fungal isolate, and its sequence has been submitted to a gene database under the same accession number, as illustrated in Figure 2.

#### GC-MS Analysis

GC–MS is an effective combination for chemical analysis. GC analysis separates compounds in their complex mixtures, and the MS analysis determines the molecular weight and ionic fragments of individual components, further aiding in the identification of those compounds. Helium was used as the carrier gas at a flow rate of 1 mL min^−1^. The compounds were identified based on their mass spectra and using the National Institute of Standards and Technology (NIST) library. The numerous standards of the American Society for Testing and Materials that cover GC/MS are also utilized for routine determinations. The following details are shown for each chemical in Table 1. Molecular formula is the chemical formula representing the elements and their quantities in each molecule. Molecular weight (g/mol) is the weight of one mole of the compound in grams. Peak area% is the percentage of the total chromatographic peak area attributed to the compound. Compound name is the full chemical name of the compound. Retention time (RT) is the time at which the compound elutes from the chromatographic column, measured in minutes. Furthermore, Table 1 presents the results obtained from GC-MS analysis for over 90% of the identified compounds. In the biomass filtrate of *A. flavus*, the predominant compounds were hexadecanoic acid (22.97%) and its methyl ester version (9.59%). These were closely followed by a series of other compounds, including the methyl ester of octadecanoic acid (9.29%), octadecanoic acid itself (6.23%), 1,4-diaza-2,5-dioxobicyclo [4.3.0]nane (4.43%), (Z)-13-Docosenamide (3.43%), hexadecanoic acid, octadecyl ester (3.05%), (Z)-9-Octadecenamide (2.43%), and 5-Hydroxymethylfurfural (1.76%) in descending order of their content ratios and other compounds of medicinal and industrial importance listed in the tables.

### 2.2. Se-NPs Bio Fabrication

**Figure 3:**
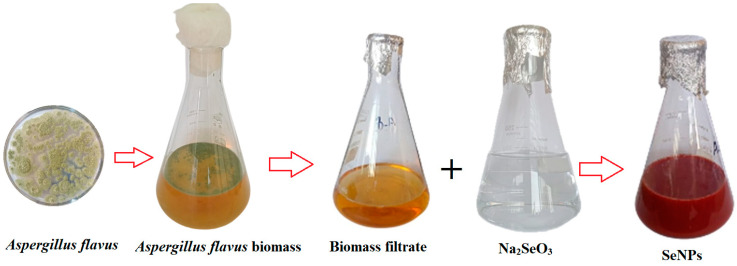
Se-NPs biosynthesized by *A. flavus*.

When the metabolites naturally present in the *A. flavus* biomass filtrate were combined with Na_2_SeO_3_, Se-NPs were formed. Compounds secreted by *A. flavus* aid in the stabilization of these nanoparticles and facilitate the reduction of selenium ions to create Se-NPs. Figure 3 clearly shows the appearance of a dark ruby red color after the interaction of sodium selenite with the *A. flavus* bio-mass filtrate, indicating the successful synthesis of Se-NPs. To finalize the process, the Se-NPs were extracted from the colloidal solution and dried in an oven at 80 °C, resulting in a deep reddish powder.

#### 2.2.1. Characterization

**Figure 4:**
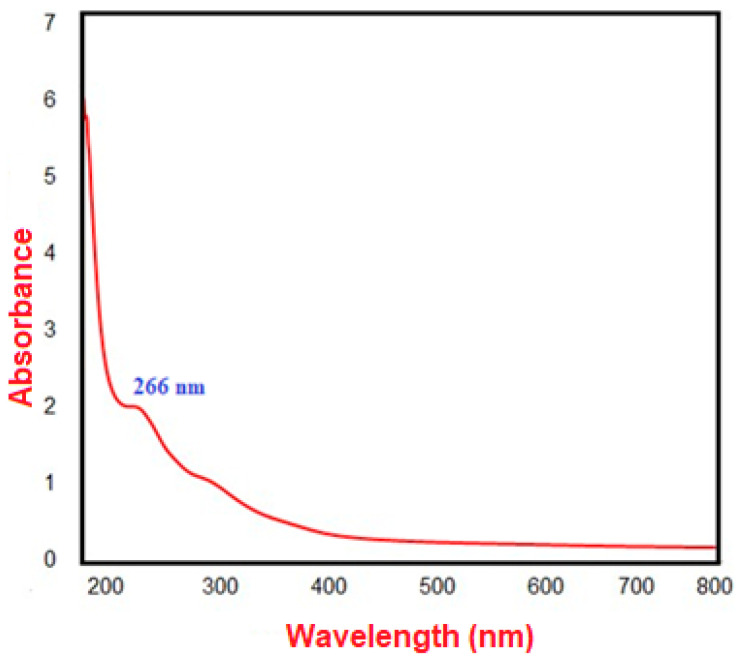
The biosynthesized Se-NPs’ UV-vis spectrophotometry.

Figure 4 illustrates that UV–visible spectroscopy confirmed the biosynthesis of Se-NPs, with the most significant absorption peak for the produced Se-NPs occurring at 266 nm. This peak is attributed to the surface plasmon resonance (SPR) of the nanoparticles. The SPR band is created by the excitation of free electrons, which is responsible for this wide peak in the visible spectrum. This excitation is facilitated in nanoparticles like Se-NPs due to the proximity of the valence and conduction bands.

**Figure 5:**
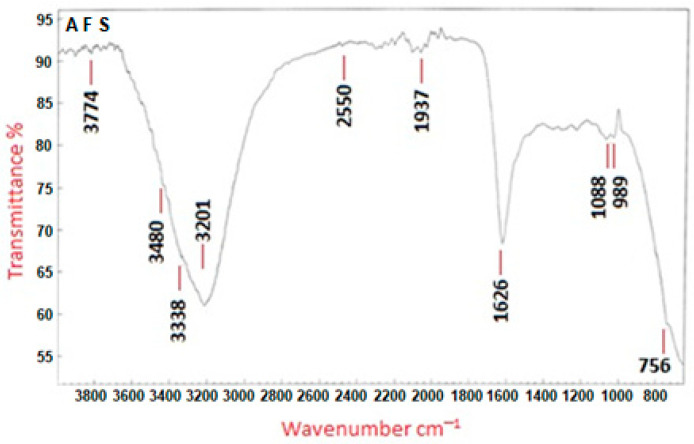
FT-IR spectra of Se-NPs produced by *A. flavus.* AFS expresses *A. flavus* biosynthesized Se-NPs.

FTIR analysis was instrumental in detecting various functional groups present in the biomaterials that play a key role in the synthesis, capping, and stabilization of the biosynthesized Se-NPs. This was achieved by matching the observed spectral bands with known reference spectra. According to Figure 5, the absorption peaks found at wavenumbers 3774, 3480, 3338, 3201, 2550, 1937, 1626, 1088, 989, and 765 cm^−1^ are indicative of the interaction between the capping agent and the biosynthesized Se-NPs. The peaks at 3774, 3480, 3338, and 3201 cm^−1^ suggest the presence of O-H stretching vibrations from phenols and alcohols, as well as possibly N-H asymmetric stretching from amines. The peak at 2550 cm^−1^ could denote the presence of alkyne triple bonds (C≡C) or alkene double bonds (C=C). The absorption at 1626 cm^−1^ may correspond to the amide I band vibrations of proteins, which include N-H stretching, or indicate aromatic compounds like polysaccharides. The peak at 1088 cm^−1^, indicative of N-C and C-C stretching, points to protein presence. Importantly, the peak observed at 765 cm^−1^ in the FT-IR spectra of the biosynthesized Se-NPs is linked to the Se-NPs’ interaction with biological materials derived from the *A. flavus* filtrate. These findings imply the involvement of proteins in stabilizing the nanoparticles through their interaction with the Se-NPs.

**Figure 6:**
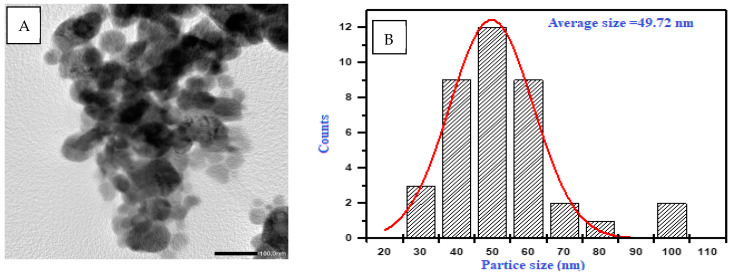
TEM images (**A**) and DLS analysis (**B**) of the biosynthesized Se-NPs.

Transmission electron microscopy was employed to ascertain the size and morphology of the Se-NPs. The TEM investigation, shown in Figure 6A, confirmed that the Se-NPs produced with *A. flavus* biomass filtrate had a spherical form. These nanoparticles exhibited a tight size distribution and were encapsulated by bioactive metabolites. The size of the biosynthesized Se-NPs predominantly ranged between 28 and 78 nm, with the average particle size being estimated through dynamic light scattering. As depicted in Figure 6B, the mean hydrodynamic diameter of the biosynthesized Se-NPs was found to be around 50 nm.

**Figure 7:**
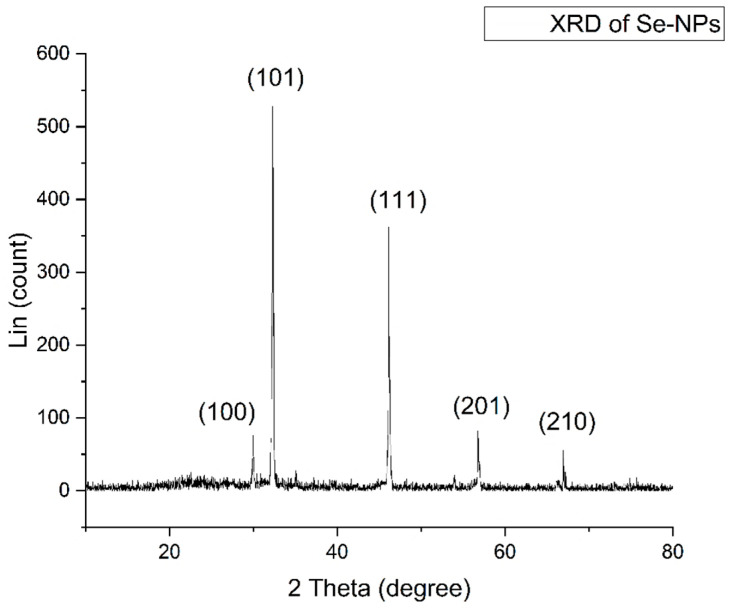
XRD assay of biosynthesized Se-NPs.

X-ray diffraction analysis offered additional information on the structural characteristics of the biosynthesized Se-NPs, as illustrated in Figure 7. The observed diffraction peaks at 2θ angles of 29.90°, 32.31°, 46.14°, 56.76°, and 66.94° matched the Bragg reflections from the (100), (101), (111), (201), and (210) planes, respectively. This suggests that the biosynthesized Se-NPs have a monoclinic crystalline structure. Using Scherrer’s formula, the average crystalline size of the biosynthesized Se-NPs was calculated, revealing that the nanoparticles were 50 nm in size based on the XRD analysis.

#### 2.2.2. The Cytotoxic Effect

**Figure 8:**
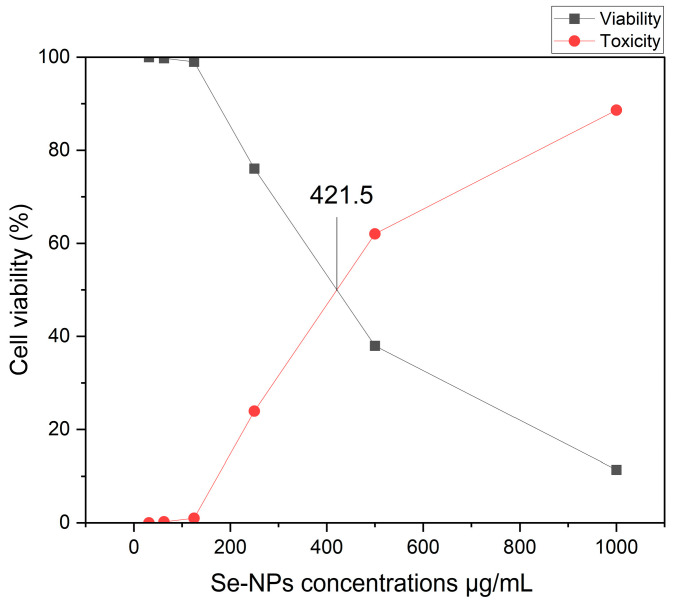
Se-NPs’ impact on healthy Vero cells.

The earliest and most notable observation following exposure to nanoparticles, or any other harmful compounds is either an alteration in the form or morphology of the cells in culture. Therefore, depending on the dose of Se-NPs exposure, the light inverted microscope might be utilized to track changes in cell morphology and shape. The normal cell line had a classic epithelial shape and spread continuously over the plate. The phenotypic characteristics of the cell were gradually lost as it was subjected to varying quantities of Se-NPs. Figure 8 displays the cytotoxic impact of the biosynthesized Se-NPs on the Vero cell line, with the half-maximal inhibitory concentration (IC50) evaluated over Se-NP concentrations ranging from 1000 to 31.25 μg/mL. The findings revealed that the IC50 value for Se-NPs produced via fungal synthesis was established at 421.52 ± 0.93 μg/mL. The normal Vero cell showed 100% viability at 31.25 μg/mL, and viability decreased by increasing Se-NPs concentrations.

### 2.3. Anticancer Activity

**Figure 9:**
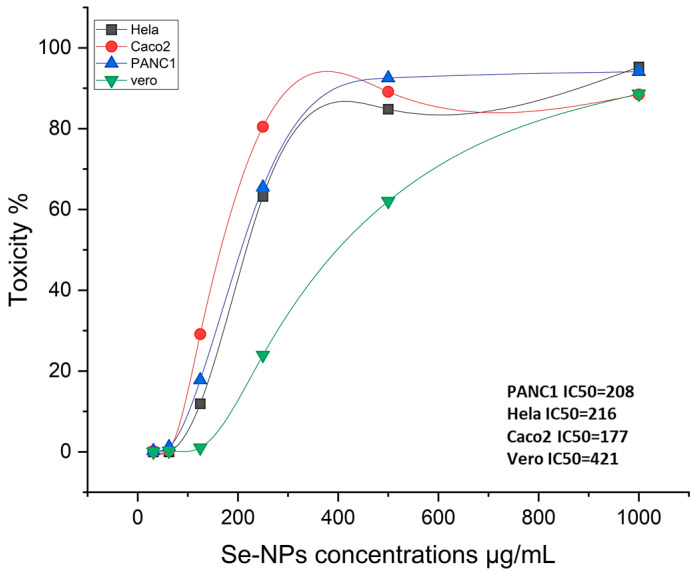
Cytotoxic and anticancer effect of the biosynthesized Se-NPs.

We evaluated the anticancer efficacy of Se-NPs biosynthesized through fungal processes against three cancer cell lines, with doses ranging from 31.25 to 1000 μg/mL. Figure 9 showcases the impact on human colorectal adenocarcinoma epithelial cells, pancreatic carcinoma cells, and cervical cancer cells. Data represented in Supplementary Figure S1 revealed that cell mortality for normal and cancer cells was also dose-dependent when exposed to different concentrations of the biosynthesized Se-NPs. Under a light-inverted microscope, normal cells exhibit a typical epithelial appearance and spread automatically. Exposure to varying quantities of Se-NPs causes the gradual loss of these normal phenotypic traits. The reactivity of released metal ions with cellular constituents may be the source of Se-NPs’ cytotoxicity. This process produces reactive oxygen species (ROS), which exacerbate oxidative stress and ultimately result in apoptosis. Additionally, this connection prevents cell repair by reducing ATP generation and harming enzymes that are essential for the functioning of proteins in the cell, such as protein kinases. The results indicated that the biosynthesized Se-NPs exhibited promising anticancer activity, particularly against Hela cells, with an IC50 value of 216 ± 1.87 μg/mL. Moreover, the IC50 values determined for PANC1, and Caco-2 cells were 208 ± 1.22 μg/mL and 177 ± 1.62 μg/mL, respectively. Further microscopic observation of these cells treated with IC50 concentrations of Se-NPs revealed unhealthy cells that lost their typical shape morphology, had a partial or complete loss of monolayer, rounding, shrinking, or cell granulation when compared to untreated control cells, which showed a well-organized cytoskeleton, as detailed in the biological process illustrated in Figure S1. These findings suggest that Se-NPs, when biosynthesized via fungal methods, show potential anticancer properties, and could be explored further as anticancer agents.

### 2.4. Antibacterial Assay

In this study, the antimicrobial efficacy of the biosynthesized Se-NPs was assessed using the well diffusion method against various infectious bacteria. The Se-NPs, produced by the cell-free filtrate of *A. flavus*, demonstrated considerable antimicrobial activity, particularly against *B. subtilis*, *S. typhimurium*, and *C. sporogenes*, with inhibition zones measuring 31 ± 0.2, 29.1 ± 0.7, and 29.1 ± 0.3 mm, respectively. In contrast, *S. aureus*, *E. coli*, and *B. pumilus* were more resistant, exhibiting inhibition zones of 28.2 ± 0.3, 27.9 ± 0.4, and 25.5 ± 0.4 mm. The control tests, negative (DMSO), did not show clear zones of inhibition, while the positive (standard antibiotic) showed moderate inhibition activity toward these pathogenic bacteria (Figure S2). This result highlights the potential of the biosynthesized Se-NPs as an effective antibacterial agent against moderate bacterial strains. The study reported an approximate 100% average growth inhibition percentage across all bacterial strains, as detailed in Table 2 by the broth microdilution test.

Also, we investigated the inhibitory effects of Se-NPs at various concentrations (16.62–1000 µg/mL) to determine the MIC. The results showed that the MIC for the biosynthesized Se-NPs against *B. subtilis* was 62.5 µg/mL while, for *S. typhimurium* and *C. sporogenes*, the MIC was 125 µg/mL for both. On the other hand, the MIC values for *S. aureus* and *E. coli* were found to be 500 µg/mL, while for *B. pumilus*, it was determined to be 1000 µg/mL, as depicted in Figure S3 and Table 2.

### 2.5. Anti-Biofilm Activity

**Figure 10:**
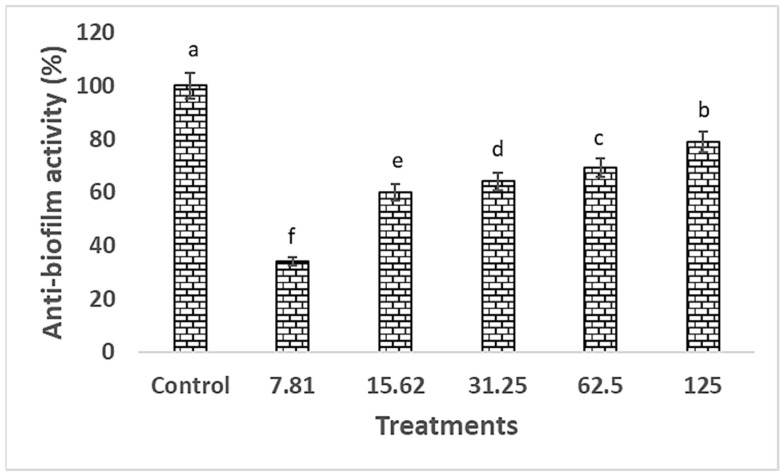
Antibiofilm inhibition percent of Se-NPs biosynthesized by using *A. flavus* filtrate against MRSA at different concentrations. Control is MRSA biofilm formation and different letters (a, b, c, d, e and f) on bars at the same concertation denote that mean values are significantly different (*p* ≤ 0.05) (n = 3).

In this study, the ability of nanoparticles to disrupt biofilm formation by MRSA varied, with Se-NPs demonstrating significant antibiofilm activity. Specifically, the biosynthesized Se-NPs were most effective at preventing MRSA biofilm formation when used at concentrations below their MIC, with doses of 15.62, 31.25, 62.5, and 125 μg/mL reducing biofilm formation by 34.0%, 60.0%, 64.0%, 69.2%, and 78.8%, respectively, as shown in Figure 10. The biosynthesized Se-NPs were found to interfere with the initial stages of MRSA biofilm development, according to both quantitative and qualitative assessments. This disruption was enhanced by the synergistic action of conjugated Se-NPs, further reducing the biofilm-forming capacity of MRSA.

### 2.6. Antioxidant Activity

**Figure 11:**
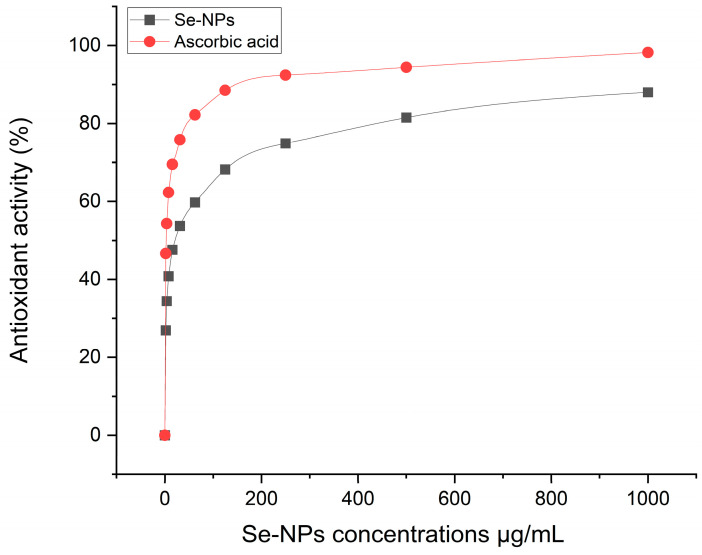
Antioxidant activity of the biosynthesized Se-NPs and ascorbic acid as reference at different concentrations.

Reactive oxygen species (ROS), which are produced through biological processes and can oxidatively damage cellular components, often lead to cell death. To mitigate the harmful impact of ROS, antioxidant compounds are frequently utilized. In this study, the DPPH free radical assay was employed to evaluate the antioxidant potential of Se-NPs biosynthesized through fungal processes. Using ascorbic acid as a benchmark for comparison, Figure 11 demonstrates the antioxidant activities of Se-NPs across different concentrations. The results revealed that the biosynthesized Se-NPs showed significant antioxidant capacity, with an IC50 value of 20.39 μg/mL against the IC50 value of 2.8 μg/mL of ascorbic acid.

### 2.7. Antiviral Activity

**Figure 12:**
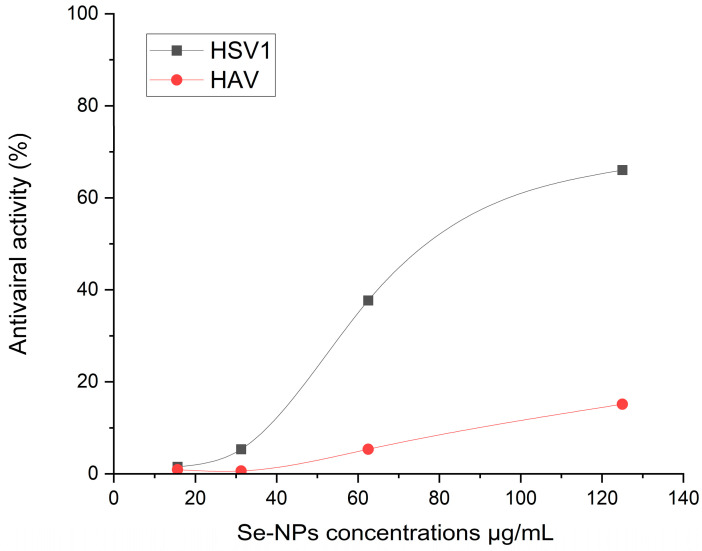
Antiviral activity % of the biosynthesized Se-NPs against human viruses, HSV1 and HAV at different concentrations.

Antiviral activity in a scientific context typically refers to the complete inhibition or neutralization of a virus by an antiviral agent under specific conditions. This means that the antiviral agent is fully effective at preventing the virus from infecting cells, replicating, or causing disease in the tested system. Studies have found that a deficiency in selenium can significantly increase susceptibility to viral infections. One of the advantages of using Se-NPs for their antiviral properties includes their lower toxicity and enhanced effectiveness. This highlights the importance of further research into the development of novel nanomaterials that incorporate antiviral compounds. As depicted in Figure 12, the antiviral capabilities of the biosynthesized Se-NPs were tested against human viruses, namely HSV1 and HAV. The maximum non-toxic concentration of Se-NPs, established at 125 µg/mL, was assessed using a standardized Vero cell line and was found to be significantly effective against these viruses. The biosynthesized Se-NPs showed promising antiviral efficacy against both HSV1 and HAV, with HSV1 being more susceptible. Specifically, the antiviral activity of Se-NPs against HSV1 was recorded at 66.04%, while it was 15.1% against HAV at the same concentration (125 µg/mL). These findings demonstrate the potential of the biosynthesized Se-NPs as effective antiviral agents against both HAV and HSV1, underscoring their potential applications in biological contexts.

## 3. Discussion

In the present study, colorectal cancer (CRC) ranks as the third most common cause of cancer-related mortality, with its incidence increasing in developed countries [26]. Therefore, it is critical to discover efficient methods for managing and treating colon cancer. The biosynthesized nanoparticles are one of the novel approaches to fighting cancer, particularly colon cancer. To explore this, we produced Se-NPs utilizing the fungal-free filtrate of *A. flavus*. This filtrate acted as both a reducing and a stabilizing agent for the nanoparticles.

Furthermore, reductive proteins and enzymes that fungi exude into their environment convert selenium ions into solid, non-toxic Se-NPs [27]. In our study, the peak absorption of the produced Se-NPs was identified at 266 nm. Similarly, Fouda et al. [17] found that Se-NPs synthesized with *Portulaca oleracea* extract showed a peak of maximum absorbance at 266 nm. These data emphasize how well fungal extracts work as biocatalysts to convert SeO_3_^2−^ to Se^0^. Bafghi et al. [28] showed that the maximal surface plasmon resonance (SPR) peak of Se-NPs was seen at 295 nm (λmax) and 310 nm (λmax), respectively, indicating that both *A. flavus* and *C. albicans* are capable of manufacturing Se-NPs using a green and ecologically friendly procedure. *Penicillium expansum* has also been shown by Hashem et al. [29] to be able to produce Se-NPs. Various bacteria have been shown to convert inorganic selenite (SeO_3_^2−^) or selenate (SeO_4_^2−^) into elemental selenium (Se^0^) nanoparticles, which can take on different shapes, including spherical, hexagonal, polygonal, and triangular [30]. Additionally, it has been noted that biogenic Se-NPs made with *Clausena dentata* leaf extract have strong, dose-responsive mosquito larvicidal properties [31].

The transformation of the yellow fungal extract into a dark ruby red color indicates the successful use of *A. flavus* extract as a reducing agent in the biosynthesis of Se-NPs, with the change in color serving as an indicator of nanoparticle formation in various studies [29,32]. The transition to a dark ruby red color is ascribed to the excitation of surface plasmon vibrations in the nanoparticles, a phenomenon confirmed by observing a UV-Vis absorption peak [33]. Jha et al. [34] found that the UV–visible spectrum of RMLP-SeNPs peaked at 265 nm. Similarly, Saied et al. [35] detected two absorption peaks at 218 and 284 nm in Se-NPs synthesized by *A. terreus*. The size of nanoparticles is crucial for their biological applications, with smaller nanoparticles often having more significant effects on biological properties [36]. Research has indicated that smaller Se-NPs are particularly effective in stopping the growth of tumor cells through a process mediated by ROS [14].

The results of transmission electron microscopy images revealed that the biosynthesized Se-NPs were spherical, with diameters ranging from 28 to 78 nm. Saied et al. [35] reported that *A. terreus* was used for the synthesis of Se-NPs ranging in size from 10 to 100 nm. Jha et al. [34] conducted a study whereby they observed that the optimized RMLP-SeNPs (which were made from polysaccharide RMLP from *Rhizophora mucronata* leaves) had a size distribution of around 31.82 ± 8.46 nm when observed through high-resolution TEM (HR-TEM). Dynamic light scattering analysis indicated that the optimized average particle size of RMLP-SeNPs was around 54.85 ± 0.55 nm, with a low Polydispersity Index (PDI), indicating a uniform distribution of nanoparticle sizes [34].

Fourier-transform infrared spectroscopy was employed to examine the interactions of functional groups with selenium, which is crucial for the formation and stabilization of Se-NPs [37]. These findings suggest that proteins binding to Se-NPs could contribute to their stabilization. It is crucial to understand that the significance lies not just in the size and shape of proteins but also in their molecular conformation within this context. Proteins can attach to nanoparticles via their free amine groups or cysteine residues [38]. Furthermore, proteins found in the fungal extract might engage with the metal nanoparticles through their free amino or carboxyl groups [39].

X-ray diffraction techniques were applied to assess the crystalline nature and structural details of the nanoparticles [40]. A study by Jha et al. [34] discovered in their research that the XRD pattern of the RMLP-SeNPs they synthesized showed peaks at 2θ values of 22.97, 29.11, 40.76, 43.22, 44.82, 51.08, 55.23, 61.70, 64.89, and 68.16, each matching different crystalline planes. Similarly, Saied et al. [35] observed five specific peaks in the XRD pattern of Se-NPs produced using *A. terreus*, which corresponded to the crystalline planes of (100), (101), (111), (201), and (210).

Different peaks were observed, and coupled gas chromatography–mass spectrometry was employed to identify the larger components. Seventeen peaks were observed in Table 1 for many compounds, such as hexadecanoic acid, octadecanoic acid and 1,4-diaza-2,5-dioxobicyclo [4.3.0] nane, (Z)-13-Docosenamide, hexadecanoic acid, octadecyl ester, (Z)-9-Octadecenamide, 5-Hydroxymethylfurfural and its methyl ester version. In another study reported by Kaminśki et al. [41], who observed nineteen peaks of *A. flavus* for large compounds such as 3-methyl-butanol, 3-octanone, 3-octanol, 1-octen-3-ol, 1-octanol, and cis-2-octen-1-ol. Abdelghany et al. [42] used GC/MS analysis of *J. procera* extract and observed 46 constituents related to different secondary metabolites. Hexadecanoic acid methyl ester is a type of fatty acid ester [43]. It can inhibit the growth of pathogenic bacteria. Hexadecanoic acid methyl ester exhibited antibacterial potency against *S. aureus* W35, *P. aeruginosa* D31, *K. pneumoniae* DF30, and *K. pneumoniae* B45 [44]. The important compounds of 9-octadecenoic acid (Z), and n-hexadecanoic acid are stated to have antioxidant, antibacterial, anticancer, hepatoprotective, and anti-inflammatory properties [45]. 9,12-octadecadienoic acid, methyl ester (linoleic acid), which is used as a hypocholesterolemic, anticancer and anti-inflammatory agent, among others, and 1-octadecene (which has an important role in nanoelectronics production) [46] are materials that are very useful in reducing selenium ions and forming nano-selenium, which has gained a lot of effectiveness, such as in anticancer, antiviral, antibacterial and antioxidant activities.

The fundamental step in toxicology that explains the cellular reaction to a toxin is called a viability assay. They also provide details on metabolic processes, cell survival, and death [47]. It is interesting to note that nanoparticles produced naturally have more activity than those created chemically or physically [48]. MTT is a reliable colorimetric screening technique that is frequently used to gauge the toxicity and viability of cells. Our findings from the MTT assay revealed significant antitumor effects on three cancer cell types: PANC1, Hela, and Caco-2, showing IC50 values of 177, 208, and 216 μg/mL, respectively. It was noted that cell viability decreased as the concentration of Se-NPs increased. Biologically produced Se-NPs caused comparable morphological alterations in the normal Wi 38 and malignant Caco-2 cell lines, causing the cells to shrink, round, or granulate in addition to losing their shape and monolayer integrity [49]. Meanwhile, Thamer et al. [50] showed a marginal effect of CuO-NPs biosynthesized using *Cordia myxa* L aqueous extract on the viability of human normal cells at concentrations up to 100 μg/mL. Fouda et al. [17] explored the anticancer effects of biosynthesized Se-NPs on human normal lung fibroblast (WI-38) and human hepatocellular carcinoma (HepG2) cells across a range of concentrations (32.25–1000 µg·mL^−1^) over a 24 h period. Their research revealed that Se-NPs induced cytotoxic effects on both WI-38 and HepG2 cells in a concentration-dependent manner. Keshtmand et al. [32] found that SeNPs were cytotoxic to HT-29 colon cancer cell lines at all tested concentrations after 24 and 48 h. The Se-NPs were less harmful to normal cells (HEK293), indicating that the nanoparticles impacted the survival of both cancerous and normal cells in ways that depended on the dose and duration of exposure. Numerous studies have highlighted the anticancer potential of phyto-synthesized nanoparticles against various cancer cell lines, exhibiting dose-dependent efficacy [51,52]. Another study highlighted the cytotoxic potential of Se-NPs in prostate cancer treatment through the induction of cell cycle delay and stimulation of cell death in prostate cancer cells [53]. Tabibi et al. [54] discovered that elevated levels of synthesized Se-NPs were effective in suppressing the proliferation of HT-29 and MCF-7 cancer cell lines. Its cytotoxicity can be explained by the Se-NPs diffusing into the cell membrane through ion channels, where they come into contact with intracellular proteins or the nitrogen bases of DNA to induce cell cycle arrest, mitochondrial malfunction, DNA fragmentation, and apoptosis [29,55].

Our investigation into the antimicrobial effects of the biosynthesized Se-NPs revealed their efficacy against a range of bacterial strains, demonstrating inhibition zones of 31 ± 0.2, 29.1 ± 0.7, 29.1 ± 0.3, 28.2 ± 0.3, 27.9 ± 0.4, and 25.5 ± 0.4 mm. The results from the MIC test further verified the substantial antibacterial effectiveness of Se-NPs against both Gram-positive and Gram-negative bacteria. The variation in antibacterial activity among different pathogenic bacteria is probably attributed to the differences in their cell wall structures. The success of Se-NPs in combating various Gram-positive and Gram-negative bacteria implies that Gram-positive bacteria may possess a greater membrane surface charge. Jha et al. [34] indicated that the RMLP-SeNPs they created showed antimicrobial action against *S. aureus*, *P. aeruginosa*, *E. coli*, and *Aeromonas hydrophila*. The antimicrobial effectiveness of RMLP-SeNPs, alongside Se-NPs, Na_2_SeO_3_, and a positive control, was assessed based on the inhibition zones against pathogens like *A. hydrophila*, *S. aureus*, *E. coli*, and *P. aeruginosa* at a concentration of 100 μg/mL. Previous findings have linked the antimicrobial properties of Se-NPs to their shape; spherical nanoparticles, for instance, are more readily internalized by cell compartments due to their interaction with cell membranes, compared to larger, elongated particles [20]. Hashem et al. [56] biosynthesized Se-NPs using *Urtica dioica* (stinging nettle) leaf extract with sizes ranging from 21.7 to 83.6 nm showed minimal inhibitory concentration (MIC) of SeNPs against *Escherichia coli*, *Pseudomonas aeruginosa*, *Bacillus subtilis*, and *Staphylococcus aureus* were 250, 31.25, and 500 μg mL^−1^, respectively, while 62.5, 15.62, 31.25, and 7.81 μg mL^−1^ against *Candida albicans*, *A. fumigatus*, *A. niger*, and *A. flavus*, respectively. In another study, SeNPs produced by using *Annona muricata* fruit aqueous extract were observed against Gram-positive bacteria related to Gram-negative bacteria. The zone of inhibition against Gram-positive bacteria was observed in the range of 09.17 ± 0.23–23.41 ± 0.50 mm [57]. Hashem et al. [29] illustrated that Se-NPs have potential antimicrobial activity against Gram-positive (*Bacillus subtilis* ATCC6051 and *S. aureus* ATCC23235), Gram-negative bacteria (*E. coli* ATCC8739 and *Pseudomonas aeruginosa* ATCC9027), and fungi (*C. albicans* ATCC90028, *A. niger* RCMB 02724 and *A. fumigatus* RCMB 02568). The results of the study showed that 2000 μg/mL of Se-NPs had an inhibition zone diameter of 36.3 ± 0.882 mm for *S. aureus* ATCC23235 and *B. subtilis* ATCC6051, 30.3 ± 1.093 mm for *E. coli* ATCC8739, 28.3 ± 0.333 mm for *B. subtilis* ATCC6051, 26 ± 0.557 mm for 25.6 ± 0.667 mm for *A. fumigatus* RCMB 02668, and 22.9 ± 0.493 mm for *A. fumigatus* RCMB 02724. Mosallam et al. [58] synthesized poly-dispersed SeNPs with an average particle size of 55.0 nm by using *A. oryzae* and showed activity towards *Acinetobacter calcoaceticus* (15.0 mm ZOI) and *S. aurus* (16.6 mm ZOI). Additionally, SeNPs inhibited *C. albicans* (15.3 mm ZOI) and mycotoxin producing *A. flavus* (29.6 mm ZOI). Se-NPs produced using *Stenotrophomonas maltophilia* SeITE02, which is smaller than 100 nm, has exhibited enhanced antimicrobial effects against *S. aureus*, *E. coli*, and *P. aeruginosa*, in comparison to those with sizes ranging from 100 to 400 nm [59]. The pronounced antimicrobial activity of Se-NPs, particularly their ability to release selenium ions that disrupt bacterial structures, positions them as a viable alternative to combat multidrug-resistant bacterial infections [60]. Additionally, chitosan-enhanced Se-NPs have shown effective, dose-dependent inhibition against *C. albicans* biofilms, underscoring the fungicidal potential of Se-NPs [61]. The mechanism behind the inhibitory impact of Se-NPs obtained from fungus is probably associated with the generation of ROS, which impedes the integrity of the plasma membrane and interferes with selective permeability. Moreover, these biosynthesized Se-NPs may have an antimicrobial effect by rupturing the cell wall, adhering to the membrane, entering the cytoplasm, and causing modifications or mutations in DNA replication, metabolic cycles, protein synthesis, and interactions with thiol or sulfhydryl groups in amino acids and proteins, which results in denaturation and cell death [62,63].

Approximately 65% of infections are attributed to biofilm-producing bacteria, with *P. aeruginosa* and *S. aureus* being among the most prevalent strains [64]. Our investigation revealed that the biosynthesized Se-NPs were most effective at preventing MRSA biofilm formation when used at concentrations below their MIC, with doses of 15.62, 31.25, 62.5, and 125 μg/mL reducing biofilm formation by 34.0%, 60.0%, 64.0%, 69.2%, and 78.8%, respectively. Jha et al. [34] assessed the capacity of RMLP-SeNPs to inhibit biofilm development by different pathogens at concentrations varying from 10 to 100 μg/mL. At the minimal concentration of 10 μg/mL, the RMLP-SeNPs demonstrated an antibiofilm effect, reducing biofilm formation by 2.87 ± 1.24% to 13.14 ± 1.47% across all tested pathogens. According to one study, when applied to *S. aureus*, *P. aeruginosa*, and *P. mirabilis*, Se-NPs with particle sizes between 80 and 220 nm were shown to suppress biofilm formation by 42%, 34.3%, and 53.4%, respectively [65]. Se-NPs, synthesized from *B. licheniformis* TUB5, with diameters ranging from 10 to 50 nm, were shown to be extremely efficient antibiofilm agents against bacterial strains, including *E. faecalis*, *S. aureus*, *E. coli* O157:H7, *S. typhimurium*, and *S. enteritidis* [66]. Additionally, biogenic Se-NPs produced from *B. licheniformis* JS2 successfully prevented the adhesion and biofilm formation of *S. aureus* colonies on various polymeric surfaces [67].

The study demonstrates that the biosynthesized Se-NPs possess notable antioxidant properties, indicated by an IC50 value of 20.39 μg/L, reflecting their capacity to neutralize free radicals. The DPPH assay used in the study underscores the ability of Se-NPs to reduce DPPH radicals, transforming them into a non-radical form, thereby demonstrating their free radical scavenging capability [8]. Comparatively, smaller Se-NPs are more effective antioxidants than larger ones. Research by Jha et al. [34] noted that the DPPH radical scavenging effectiveness of RMLP-SeNPs, Se-NPs, and vitamin C reached 75.45%, 66.21%, and 86.10%, respectively, at their highest tested concentration of 200 μg/mL.

Se-NPs showed good antiviral activity against HSV1 and HAV in our data, with HSV1 being more sensitive. Specifically, the antiviral activity of the biosynthesized Se-NPs against HSV1 was recorded at 66.04%, while it was 15.1% against HAV at the same concentration (125 μg/mL). Beyond just killing viruses, its antiviral qualities also involve controlling the actions of selenoproteins, making Se-NPs potentially effective agents with a wide range of antiviral activities [68]. Se-NPs have been shown to prevent the H1N1 virus from infecting MDCK cells and inducing apoptosis by blocking chromatin condensation and DNA fragmentation. This mechanism includes the reduction in ROS generation and the initiation of p53 phosphorylation and Akt activation. These actions contribute to their protective impact on cell and lung tissue damage induced by the H1N1 virus by altering apoptotic signaling pathways [69]. Additionally, Lin et al. [70] created a Se-NPs system with siRNA directed targeting of the EV71 VP1 gene. This system demonstrated significant efficiency in interfering with viral activities in the SK-N-SH nerve cell line, preventing infection, and reducing apoptosis in host cells triggered by EV71 [71]. In discussing the mechanisms of action of the biosynthesized Se-NP, it is important to note that our interpretations are based on the existing literature. Further comprehensive studies are warranted to thoroughly evaluate the antiviral mechanisms associated with the biosynthesized Se-NP. Therefore, we recommend that future investigations focus on elucidating the specific mechanisms underlying the antiviral actions of the biosynthesized Se-NP to enhance our understanding and potential applications in combating viral infections.

## 4. Materials and Methods

In this study, the analytical-grade materials employed, including sodium hydroxide and sodium selenite, an inorganic compound with the chemical formula Na_2_SeO_3_, were sourced from Sigma-Aldrich, Heliopolis, Cairo, Egypt. Sodium selenite served as the precursor for the biosynthesis of Se-NPs. All cultural media used in this research were supplied by HiMedia, Talbiya Faisal, Giza, Egypt. In the current study, distilled water (H_2_O) was used for all biosynthetic steps.

### 4.1. Fungal Isolation and Identification

The *A. flavus* used in this study was obtained from an agricultural sample and subsequently purified on a potato dextrose agar medium [72]. A pure fungal isolate was identified using genetic techniques. In summary, DNA extraction was carried out using the Thermal Gene Jet Plants Genome-wide DNA Purification Kit. The internal transcribed spacer (ITS) region was amplified using polymerase chain reaction {PCR} with primers for ITS1 (5′-TCCGTAGGTGAACCTGCGG-3′) and ITS4 (5′-TCCTCCGCTTATTGATATGC-3′). The extracted fungal genomic DNA served as the template [73].

The PCR assay, with a total volume of 50 μL, consisted of one milliliter of fungal genomic DNA, 0.5 mL for each type of primer, and Thermo’s Hot Start PCR Master Mix. The amplification process took place in a Sigma Technologies for Science DNA Engine Thermal Cycler, Nasr City, Cairo, Egypt, beginning with an initial denaturation step at 94 °C lasting three minutes. This initial step was followed by thirty cycles, each including denaturation at 94 °C for thirty seconds, annealing at 55 °C for thirty seconds, and extension at 72 °C for thirty seconds, culminating in a final extension period at 72 °C lasting ten minutes. DNA sequencing was carried out using the ABI 3730xl DNA Analyzer. The resulting ITS sequence data were then matched against the GenBank database through the NCBI BLAST tool for comparison.

### 4.2. Bio Fabrication of Se-NPs

#### 4.2.1. Generating the Fungal Filtrate

*A. flavus* was inoculated onto two 0.8 mm diameter disks and cultured in potato dextrose broth medium for 5 days at 28 °C ± 2 °C, with the pH adjusted to 7.0. The culture was maintained under shaking conditions at 130 rpm. After the incubation period, the collected biomass (10 g) was rinsed three times with deionized and sterilized water. The rinsed biomass was then resuspended in 100 mL of distilled water and incubated at 30 °C ± 2 °C with shaking at 150 rpm for two days. Following this, the suspension was filtered through Whatman No. 1 filter paper to remove any contaminants and unwanted cell debris. Following this initial filtration, the supernatant was then centrifuged at 15,000 rpm for ten minutes to further clarify the solution and ensure the complete removal of residual particulates. This filtrate was used to produce Se-NPs in an environmentally friendly manner.

#### 4.2.2. GC–MS Assay

The Chemical War Department, Ministry of Defense, Kilo 4.5, Cairo, Egypt, used GC-MS to analyze the chemical compositions of the metabolites found in the fungal extract. The Agilent 5975C Series GC/MSD and Agilent 7890A Gas Chromatograph were used to evaluate the analysis. For chemical analysis, gas chromatography (GC) and mass spectrometry (MS) work well together. Mass spectroscopy analysis helps identify individual components by determining their molecular weight and ionic fragmentation, whereas gas chromatography analysis separates substances into complicated combinations. The carrier gas, helium, was used at a flow rate of one milliliter per minute. The oven was set to 80 °C for two minutes, then 200 °C based on a rate of 10 °C for seven minutes, and finally 280 °C based on a rate of 20 °C/min. During this time, the injector temperature was kept at 280 °C. Mass spectra from the National Institute of Standards and Technology’s (NIST) library were used to identify the chemicals. For regular determinations, certain ASTM (American Society for Testing and Materials) standards covering GC/MS are also used [74].

#### 4.2.3. Creation of Se-NPs

Ten milliliters of a 1 mM sodium selenite solution were added to ninety mL of freshly prepared biomass filtrate. This mixture was then incubated at a temperature of 30 ± 2 °C for 24 h, with a pH maintained at 6.5 and a shaking speed of 130 rpm. Controls used in the experiment included the fungal biomass filtrate (FBF) without any metal precursor and the Na_2_SeO_3_ solution alone. After incubation, Se-NP colloids were centrifuged at 10,000 rpm for 10 min. The supernatant was discarded, and the pellet was washed and then dried at 80 °C for 48 h. The final product was collected and stored for subsequent analysis [35]. It was observed that approximately 2 g of Se-NPs, including selenium nanoparticles and fungal biomass components, were produced from every 100 mL of biomass filtrate under these optimized conditions. This approximate weight includes both the selenium nanoparticles (0.79 mg of selenium) and other biomass components.

#### 4.2.4. Se-NPs Characterization

The ultraviolet–visible spectrum, ranging from 200 to 800 nm, was utilized via a UV-VIS spectrophotometer (JASCO V-630, Hachioji, Tokyo Japan) to identify significant absorption peaks associated with surface plasmon resonance indicative of the biosynthesized Se-NPs. This was monitored regularly. Additionally, a transmission electron microscope (JEOL-2100, Akishima, Tokyo, Japan) was employed to examine the process of coating the droplets, which involved applying a droplet of the nanoparticle-containing solution onto copper grids coated with carbon, followed by vacuum drying it overnight. This process facilitated the assessment of the dimensions and morphology of the selenium nanoparticles produced. The distribution of particle sizes for the biosynthesized Se-NPs was evaluated via DLS using a Zetasizer (Zetasizer Nano ZN, Malvern Panalytical Ltd., Malvern, UK), maintaining a steady temperature of 25 °C and employing a scattering angle of 173°. Additionally, Fourier-transform infrared spectroscopy (JASCO, FT/IR-6100) was utilized to explore the functional groups within the compounds of the selenium nanoparticles synthesized by the fungus. For analysis, the biosynthesized Se-NPs were mixed with potassium bromide (KBr) and compressed into disks. These disks were then scanned across a range of 400–4000 cm^−1^ to obtain the FT-IR spectra. Additionally, the crystallographic structure of the Se-NPs was determined using X-ray diffraction (Philips, Eindhoven, The Netherlands). The XRD patterns were aligned inversely relative to a nickel-filtered Cu-Kα radiation source operating at 40 kV and 30 mA. The crystalline structure of the biosynthesized Se-NPs was examined over a 2 h period ranging from 10° to 80°.

### 4.3. Anti-Tumor and Cytotoxicity Assay

Cervical adenocarcinoma cells (HeLa), human colorectal adenocarcinoma epithelial cells (Caco-2), pancreatic carcinoma cells (PANC1), and normal Vero cells were obtained from the American Type Culture Collection (ATCC) for use in various cell cultures. To assess the cytotoxic effects of Se-NPs, we employed the 3-(4,5-dimethylthiazol-2-yl)-2,5-diphenyltetrazolium bromide (MTT) assay. In this study, cells were cultured in microtiter plates at 37 °C for twenty-four hours to form a dense monolayer. Each well was inoculated with a density of 1 × 10^4^ cells per well. After the formation of a confluent cell layer, the growth media were removed from the microtiter plates, and the cells were washed several times with a washing medium to cleanse the monolayer. The biosynthesized Se-NPs were then introduced in two-fold serial dilutions using a growth medium (RPMI medium) containing 2% fetal bovine serum. For the experiment, 100 microliters of each concentration (31.25, 62.5, 125, 250, 500, and 1000 μ/mL) were added to separate wells; three wells served as controls and received only the maintenance medium. After incubating the microtiter plate at 37 °C, we evaluated all cells for signs of cytotoxicity, such as cell rounding, shrinkage, granulation, or partial to complete disruption of the monolayer membrane. Bio Basic Canada Inc., Markham, ON, Canada provided an MTT solution (5 mg/mL in PBS), and approximately 20 µL of this solution was added to every well. To ensure thorough mixing of the MTT with the medium, the plate was placed on a shaker and agitated at approximately 150 rpm for 5 min. Subsequently, the plate was incubated with 5% CO_2_ at 37 °C for 4 h to allow complete metabolism of the MTT. Afterward, the medium was discarded. If necessary, any debris on the plate was removed by blotting it with paper towels. The MTT metabolite, formazan, was then redissolved in 0.2 mL of DMSO. To ensure complete dissolution of formazan and its mixing with the solvent, the mixture was shaken at 150 rpm for 5 min. Finally, any disturbances were filtered out at 620 nm, and the absorbance was read at 560 nm. There should be a direct correlation between the optical density and the number of cells [75,76].

### 4.4. Antibacterial Activity

The antibacterial activity of biosynthesized Se-NPs was tested against six pathogenic bacterial strains: *B. pumilus*, *S. typhimurium*, *S. aureus*, *C. sporogenes*, *B. subtilis*, and *E. coli*. The agar-well diffusion method was employed for this purpose. Each bacterial strain, previously cultured in Muller–Hinton broth, was uniformly spread across sterile Petri dishes containing Muller–Hinton agar. A sterile cork borer was used to create a 7 mm well in each plate, into which 0.1 mL of the biosynthesized Se-NPs were introduced to evaluate their antibacterial efficacy. The Petri dishes were incubated at 37 °C for 24 h, and the zones of inhibition were measured thereafter [77,78]. Chloramphenicol served as the positive control, while a DMSO solution acted as the negative control in the experiment. The plates underwent a 24 h incubation period at a temperature of 37 °C. Following the incubation, the clear zone diameters surrounding each well were measured to assess the Se-NPs’ antibacterial efficacy. This process helped establish the MIC of the biosynthesized Se-NPs necessary to prevent bacterial proliferation. To ensure precision, the experiment was conducted in triplicate.

#### 4.4.1. Assay for Broth Microdilution

The bacterial solution became turbid at a concentration equivalent to the 0.5 McFarland standard (1.5 × 10^8^ cfu/mL). A sterilized 96-well microtiter plate (MTP) was prepared by adding 100 μL of the biosynthesized Se-NPs and 100 μL of the bacterial inoculum to each well, resulting in a final concentration of 5 × 10^5^ cfu/mL per well. For control purposes, additional wells containing only bacterial suspension without Se-NPs served as growth controls for the pathogenic strains being tested. After incubating for 24 h at 37 °C, the optical density was measured at 620 nm using an ELISA microplate reader (the Sunrise TM-TECAN, Männedorf, Switzerland). The percentage reduction in growth (%) was calculated based on cell density, comparing the treated wells with the biosynthesized Se-NPs to the untreated control group. The growth reduction percentage (GR%) was calculated using the formula GR% = (CT/C) × 100, where T represents the treatment group with Se-NPs and C represents the cell percentage in the reference (control) group. Results were presented as the mean ± standard error, based on three replicates [79].

To assess the antibacterial efficacy of the developed Se-NPs against bacterial strains, the broth microdilution method was utilized. This approach allowed for the determination of the MIC of the Se-NPs. A two-fold serial dilution of the biosynthesized Se-NPs was conducted, and 5 µL of a bacterial culture, equivalent to half a McFarland standard, was added to the diluted Se-NPs. The mixtures were then incubated at 37 °C for 24 h [77]. Afterward, 5 µL of resazurin dye was added to each of the 96 wells and kept in the dark. The color changes from blue to pink after the incubation period indicates bacterial metabolic activity. The lowest concentration at which this color change occurred was identified as the MIC value. The MIC was determined by averaging three independent observations [80].

#### 4.4.2. Assay for Biofilm Inhibition

To evaluate the capacity of biosynthesized Se-NPs to inhibit or diminish bacterial biofilm formation, the microtiter plate method was applied to methicillin-resistant *S. aureus* (MRSA), a clinically significant strain known for its potent biofilm-producing capability. This study introduced several modifications to the biofilm formation protocol previously established [81]. Specifically, Se-NPs were added in varying concentrations to MTP wells containing tryptic soy broth (TSB) media supplemented with 1% glucose. The MRSA cultures were then diluted 1:100 in TSB and incubated in the MTP for 48 h at 37 degrees Celsius. During this period, the growth density (measured as optical density at 620 nm) was recorded prior to the removal of planktonic (non-adherent) cells from the wells. After evacuating the wells without disturbing the biofilms, the biofilms were fixed using 200 μL of 95% methanol for 10 min and washed three times with phosphate-buffered saline (PBS) at pH 7.4. Each well was then stained with 0.3% *w*/*v* crystal violet for 15 min at room temperature. For quantitative analysis of biofilm formation, 30% acetic acid was added to the stained wells after rinsing with distilled water. The absorbance was measured at 540 nm using a microplate reader STATFAX-USA (Awareness Technology, Inc., Palm City, FL, USA). The effectiveness of the biosynthesized Se-NPs was determined by comparing the absorbance values of treated wells against those of untreated control wells [82].

### 4.5. Antioxidant Activity

The capacity of biosynthesized Se-NPs to act as antioxidants was evaluated across a concentration range from 31.25 to 2000 μg/mL through their efficiency in neutralizing DPPH (2,2-diphenyl-1-picrylhydrazyl) radicals. A solution containing 1 mM of DPPH radicals was prepared using 95% ethanol. For the assay, 200 μL of each concentration of the biosynthesized Se-NPs was combined with 800 μL of the DPPH solution. This mixture was then allowed to react for 30 min at 25 °C in the absence of light, after which it was centrifuged for 5 min at 13,000 rpm [83]. The absorbance of each concentration was measured at 517 nm against a blank, with ascorbic acid serving as the reference standard. The following formula was used to calculate the DPPH scavenging activity (%) of both the standard and the various Se-NP concentrations to determine their antioxidant effectiveness:

The scavenging capacity of DPPH % = (A1 − A2)/A1 × 100

where A1 is the absorbance of the control and A2 is the absorbance of the sample.

### 4.6. Experiment to Evaluate the Antiviral Effects of Se-NPs on Hepatitis A Virus (HAV), Herpes Simplex Virus (HSV) and Vero Cell

Vero cells (a type of cell line that originates from the kidney cells of the African green monkey, and they are commonly used in virology for the propagation of viruses and to assess the cytotoxic effects of potential antiviral compounds) were used as normal cells and were placed at a density of 10^4^ cells per well in a 96-well flat-bottomed microtiter plate with 100 µL of growth media. The cells were then incubated overnight at 37 °C in an incubator with 5% CO_2_ to determine the maximum non-toxic concentration. Following the formation of the confluent sheet of Vero cells, the growth medium was disposed of, and the wells were twice cleaned using washing media. Following this, various quantities of Se-NPs (125, 62.5, 31.25, and 15.62 ug/mL per well) were applied to the connected cells. The biosynthesized Se-NPs were diluted twice in Dulbecco’s Modified Eagle Medium (DMSO) and examined in triplicate in 100 µL of each dilution; as a control, the cells were given simply all of the media. The plates were incubated in a humidified incubator at 37 °C in 5% CO_2_ and visualized frequently for up to 48 h, and any physical signs of toxicity, such as partial or complete monolayer destruction, roundness, cellular granulation, and membrane shrinkage, were recorded. After that, 20 µL of prepared MTT solution (5 mg mL^−1^ in phosphate-buffered saline (PBS)) was added to each well and mixed with a shaker at 150 rpm for 5 min. The plates were then incubated at 37 °C, in a 5% CO_2_ incubator for 4 h. Following incubation, the growth medium was discarded from the wells and the formazan crystals were resuspended in 200 µL of DMSO and mixed thoroughly by shaking at 150 rpm for 5 min. The optical density of each well was determined at 560 nm, and the background was subtracted at 620 nm. The maximum non-toxic concentration of the examined Se-NPs was determined from graph:

Cell toxicity % = 100 − cell viability %

To assess the effect of the biosynthesized Se-NPs on the inhibition of the hepatitis A virus (HAV), an unenveloped RNA virus from the Picornaviridae family, and the Herpes simplex virus (HSV), an enveloped DNA virus from the Herpesviridae family, infectivity, Vero cells were implemented in 96-well, flat-bottomed microtiter plates at a density of 10^4^ cells/well containing 200 µL growth medium and allowed to adhere overnight at 37 °C in 5% CO_2_. A virus suspension was incubated with non-lethal concentrations of Se-NPs (1:1, *v*/*v*) at room temperature for 1 h. After incubation, 100 µL from the viral/sample suspension was added to the well implemented with Vero cells, whereas three wells were considered non-infected cells (control) and contained Vero cells and growth media only. The plates were mixed on a shaker for 5 min at 150 rpm followed by incubation for 24 h at 37 °C in 5% CO_2_ to allow the virus to take effect. The cellular viability of infected and non-infected Vero cells was conducted using the absorbance values of formazan crystals used in the MTT reagent as described for the cytotoxicity assay. The anti-HAV and anti-HSV activities were determined by measuring the difference in the values between the optical density of infected and uninfected cellular viability [84].

### 4.7. Statistical Data Analysis

To demonstrate the importance of our research, we performed all analyses using a one-way analysis of variance (ANOVA) with SPSS version 18 software. Consequently, the data are presented as mean values ± standard deviation (SD), and *p*-values below 0.05 were considered statistically significant.

## 5. Conclusions

In the field of biological nanotechnology, the biosynthesis of Se-NPs through microbial fermentation is gaining traction due to their ecologically friendly and biocompatible character, which presents an extensive range of possible therapeutic applications. In our current study, we employed the cell-free filtrate of *A. flavus*, which acts as a capping and stabilizing agent, to synthesize a stable colloidal solution of Se-NPs. These biosynthesized Se-NPs were characterized using FT-IR, TEM, and UV-vis spectroscopy. The biosynthesized Se-NPs effectively inhibited a range of pathogens, including Gram-positive and Gram-negative strains, at various concentrations. Additionally, the biosynthesized Se-NPs demonstrated significant antitumor activity against three types of cancer cells: pancreatic carcinoma, cervical cancer, and colorectal adenocarcinoma, with IC50 values of 177, 208, and 216 μg/mL, respectively. The nanoparticles demonstrated antiviral activity against HSV and HAV, achieving maximum non-toxic concentration values of 66.4% and 15.1%, respectively. Moreover, they exhibited strong antibiofilm effectiveness against MRSA, and their antioxidant activity was demonstrated in a cell-free system and displayed non-hemolytic activity on human RBCs, highlighting their safety for medical applications.

## Figures and Tables

**Table 1:** GC mass identification of *A. flavus* filtrate.

No	RT	Compound Name	Peak Area%	Molecular Weight (g/mol)	Molecular Formula
1	5.43	9-Octadecenoic acid (Z), 3-[(1-oxohexadecyl)oxy]-2-[(1-oxooctadecyl)oxy]propyl ester	0.31	861.4	C_55_H_104_O_6_
2	6.32	Cyclohexanone, 4-[[(4-methylphenyl)sulfonyl] oxy]-	0.35	268.33	C_13_H_16_O_4_S
3	10.21	3-tert-Butylsulfanyl-3-fluoro-2-trifluoromethyl-acrylic acid methyl ester	1.06	260.25	C_9_H_12_F_4_O_2_S
4	20.99	5-Hydroxymethylfurfural	1.76	126.11	C_6_H_6_O_3_
5	31.45	3-Furanacetic acid, 4-hexyl-2,5-dihydro-2,5-dioxo	0.57	240.25	C_12_H_16_O_5_
6	40.53	1,4-Diaza-2,5-dioxobicyclo[4.3.0]Nonane	4.43	154.17	C_7_H_10_N_2_O_2_
7	48.29	Hexadecaoic acid, Methyl ester	9.59	270.5	C_17_H_34_O_2_
8	50.12	n-Hexadecanoic acid	22.97	256.42	C_16_H_32_O_2_
9	53.81	1-Dodecanol, 3,7,11-trimethyl-	1.32	228.41	C_15_H_32_O
10	54.66	Octadecenoic acid, Methyl ester	9.29	298.5	C_19_H_38_O_2_
11	56.45	Octadecanoic acid	6.23	284.5	C_18_H_36_O_2_
12	61.81	9-Octadecenamide, (Z)	2.43	281.5	C_18_H_35_NO
13	72.90	Tetraacosanoic acid, Methyl ester	0.85	382.7	C_25_H_50_O_2_
14	73.30	13-Docosenamide, (Z)	3.43	337.6	C_22_H_43_NO
15	77.62	9,12-Octadecenoic acid (Z,Z)-, 2,3-bis[(trimethyl silyl) oxy]propyl ester	0.82	498.9	C_27_H_54_O_4_Si_2_
16	85.83	Hexadecanoic acid, octadecyl ester	3.05	508.9	C_34_H_68_O_2_
17	85.90	4H-1-Benzopyran-4-one, 2-(3,4-dimthoxyphenyl)-3,5-dihydroxy-7-methoxy	1.67	462.4	C_22_H_22_O_11_

RT is retention time.

**Table 2:** Agar-well diffusion technique as well as broth microdilution test employed to evaluate Se-NPs’ antibacterial activity.

No.	Isolate Name	Antibacterial Assay of Se-NPs 100 µL		
		MICs µg/mL	Diameter of Inhibition Zone (mm)	Mean Growth Inhibition Percentage %
1	*S. aureus*	500 ± 0.32	28 ± 0.36	100 ± 0.03
2	*C. sporogenes*	125 ± 0.41	29 ± 0.32	100 ± 0.02
3	*S. typhimurium*	125 ± 0.44	30 ± 0.7	100 ± 0.021
4	*B. subtilis*	64 ± 0.14	31 ± 0.2	100 ± 0.03
5	*E. coli*	500 ± 0.28	27 ± 0.45	100 ± 0.02
6	*B. pumilus*	1000 ± 0.54	25 ± 0.49	100 ± 0.024

Mean ± SE.

## Data Availability

The data presented in this study are available on request from the corresponding author.

## Supplementary Materials

The following supporting information can be downloaded at: https://www.mdpi.com/article/10.3390/ph17070915/s1, Figure S1. Antitumor capability of selenium NPs against (1) PANC1, (2) CaCO2 and 3) HELA cancers cells by inverted microscope. Figure S2. Antibacterial activity of selenium NPs by agar-well approach. Figure S3. MIC test of the pathogenic bacteria against different concentration of Se-NPs (1000 to 16 μg/mL). Different letters (a, b, c, and d) on bars at the same concertation denote that mean values are significantly different (*p* ≤ 0.05) (n = 3).
