# Supplementary material for: Biomedical Promise of Aspergillus Flavus-Biosynthesized Selenium Nanoparticles: A Green Synthesis Approach to Antiviral, Anticancer, Anti-Biofilm, and Antibacterial Applications
Source: Pharmaceuticals (Basel). 2024 Jul 9;17(7):915. doi: 10.3390/ph17070915 (PMC11279975; doi:10.3390/ph17070915)
Supplement: Supplementary file 1 [file pharmaceuticals-17-00915-s001.zip › pharmaceuticals-3054644-supplementary.pdf]

Supplementary data

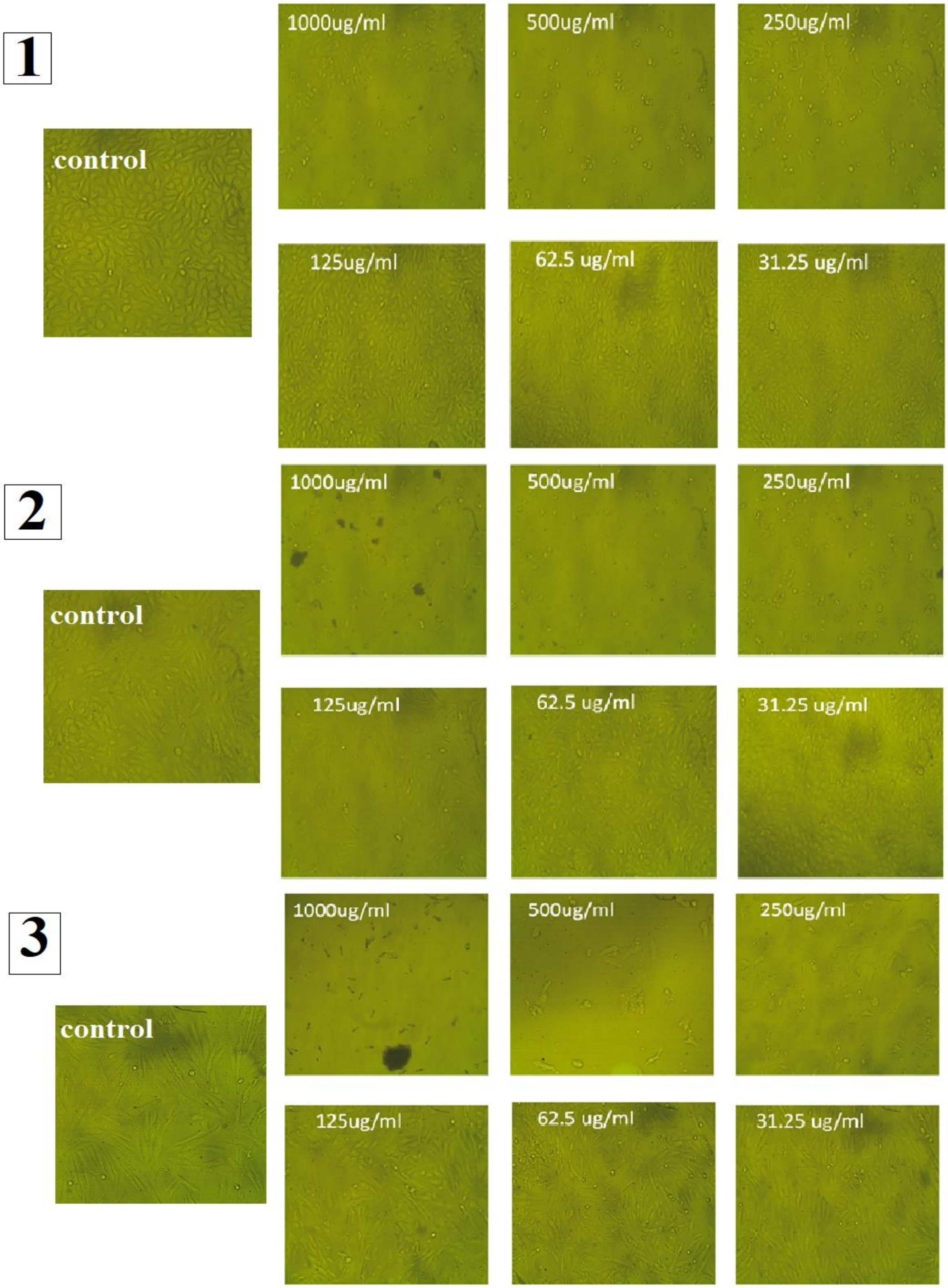

Figure S1. Antitumor capability of selenium NPs against (1) PANC1, (2) $CaCO_2$ and 3) HELA cancers cells by inverted microscope.

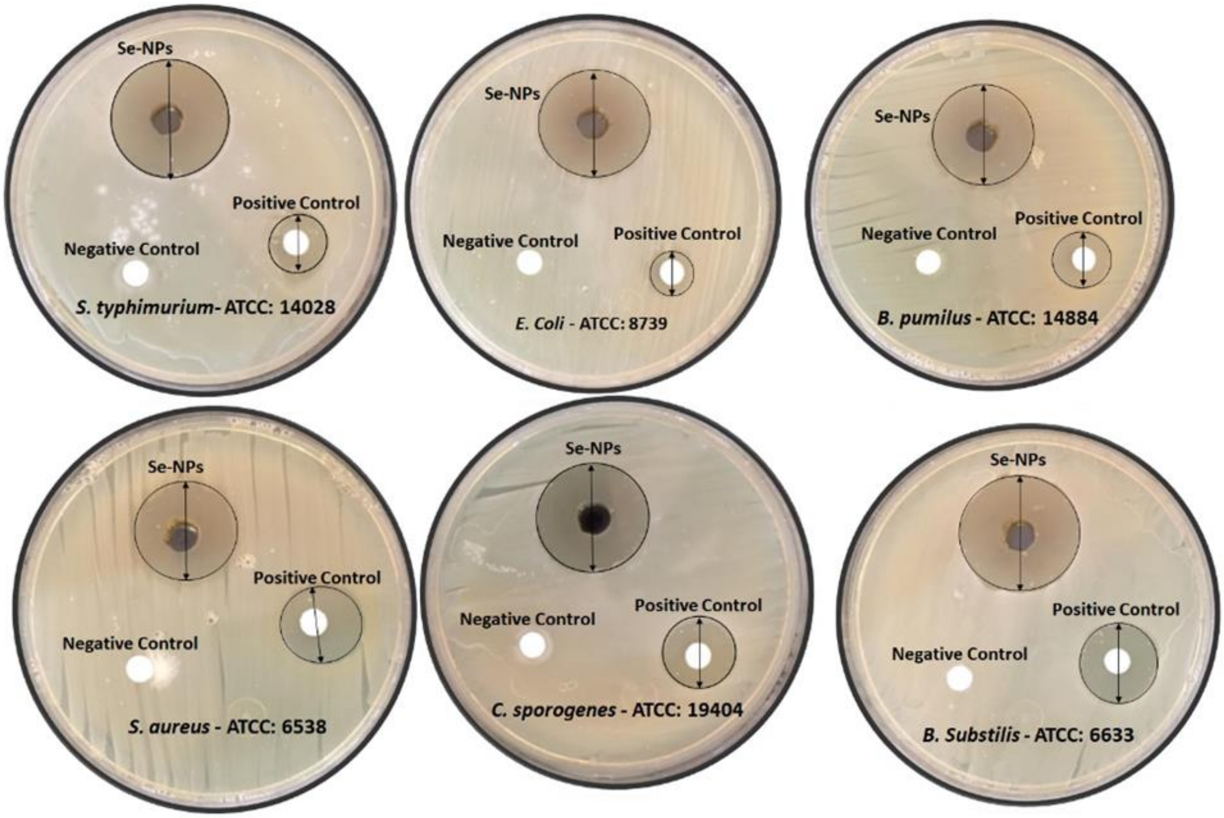

Figure S2. Antibacterial activity of selenium NPs by agar-well approach.

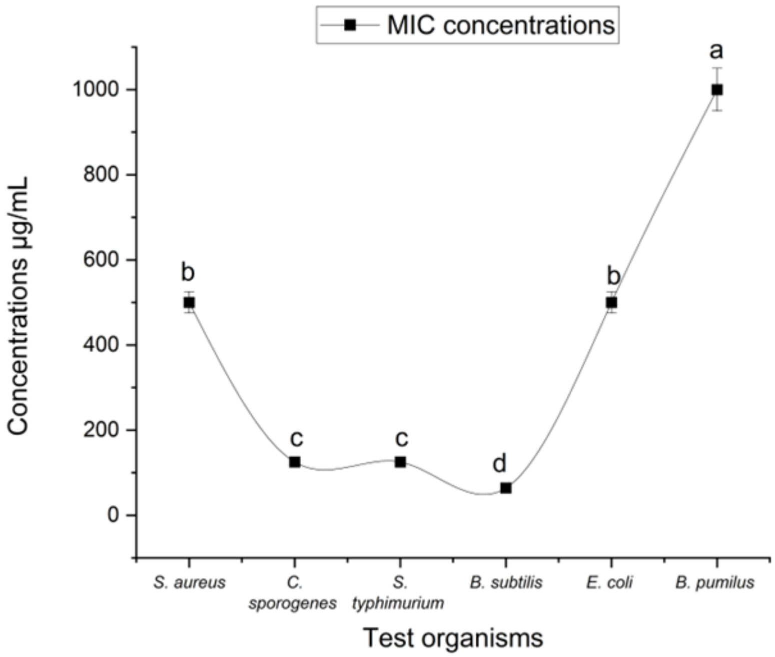

Figure S3. MIC test of the pathogenic bacteria against different concentration of Se-NPs (1000 to 16 µg/mL). Different letters (a, b, c, and d) on bars at the same concertation denote that mean values are significantly different ($p \leq 0.05$) ($n = 3$).
